# Analyses of repeated failures in cancer therapy for solid tumors: poor tumor-selective drug delivery, low therapeutic efficacy and unsustainable costs

**DOI:** 10.1186/s40169-018-0185-6

**Published:** 2018-03-01

**Authors:** Hiroshi Maeda, Mahin Khatami

**Affiliations:** 1 BioDynamics Research Foundation, Kumamoto University (Med), Kumamoto, Kenshin Bldg 3F, Kuwamizu 1-chome, 24-6, Chuo-ku, Kumamoto, 862-0954 Japan; 2 Osaka University Medical School, Osaka, Japan; 3 Tohoku University, Sendai, Japan; 4 Inflammation, Aging and Cancer, National Cancer Institute, The National Institutes of Health, Bethesda, MD USA

**Keywords:** Cancer vaccines, Cancer financial toxicity, Cancer therapeutic failure, Cancer/medical establishment, Decision makers, Enhanced permeability and retention (EPR), Genomic mutations, Immunotherapy, Incredible price of drugs, Inflammation, Medical/scientific ponzi schemes, Molecular target drugs, Molecular false flags, Nanoparticles, Oxidative stress and mutations, Precision and personalized medicine, ROS, Targeted therapy, Tarnished immune surveillance, Yin and Yang of acute inflammation

## Abstract

For over six decades reductionist approaches to cancer chemotherapies including recent immunotherapy for solid tumors produced outcome failure-rates of 90% (±5) according to governmental agencies and industry. Despite tremendous public and private funding and initial enthusiasm about missile-therapy for site-specific cancers, molecular targeting drugs for specific enzymes such as kinases or inhibitors of growth factor receptors, the outcomes are very bleak and disappointing. Major scientific reasons for repeated failures of such therapeutic approaches are attributed to reductionist approaches to research and infinite numbers of genetic mutations in chaotic molecular environment of solid tumors that are bases of drug development. Safety and efficacy of candidate drugs tested in test tubes or experimental tumor models of rats or mice are usually evaluated and approved by FDA. Cost-benefit ratios of such ‘targeted’ therapies are also far from ideal as compared with antibiotics half a century ago. Such alarming records of failure of clinical outcomes, the increased publicity for specific vaccines (e.g., HPV or flu) targeting young and old populations, along with increasing rise of cancer incidence and death created huge and unsustainable cost to the public around the globe. This article discusses a closer scientific assessment of current cancer therapeutics and vaccines. We also present future logical approaches to cancer research and therapy and vaccines.

## Background

Review of over six decades of cancer chemotherapy and tremendous investment for understanding cancer biology and cure reveal minimal or partial success for only the treatment of leukemia and non-solid or soft tissue tumors [1–7].^1^^,^^2^ The latest statistics in cancer incidence, mortality and cancer burden are growing at an alarming pace around the globe, according to governmental agencies and private organizations including the International Agency for Research on Cancer (IARC, an agency within World Health Organization (WHO), or the American Cancer Society (ACS), [8–12].^3^ In 2014, IARC reported that the global war against cancer cannot be won by treatment alone, and recommended the need for urgent implementation of efficient prevention strategies to prevent cancer crisis [8]. Clinical trials using specific cancer drugs repeatedly failed patients and the expensive therapies discontinued after loss of patients [13–16].

Other recently published articles on basic research and clinical studies of cancer and pathogen-specific vaccines have raised serious concerns about the worthiness, hidden agenda and high costs of these reductionist approaches to such projects that are toxic and repeatedly failed the public [14–40]. The majority of cancer claimed ‘targeted’ therapies, ‘personalized’ or ‘precision’ medicine are based on identification of evolving mutation-derived molecules and use of specific and expensive technologies with little or no benefit to patients. The safety and political agenda behind heavy publicity for targeting the public to consume a wide range of specific vaccines against a numbers of viruses (e.g., HPV, measles, meningitis, Ebola, Flu, Zika) are topics of debates and controversies for effectiveness of such undertaking (details below) [18, 22, 39–45].^4^^,^^5^^,^^6^

In majority of claimed cancer ‘targeted’ therapies, ‘personalized’ or ‘precision’ medicine or the recently fashionable immunotherapeutic approaches, drugs are developed as inhibitors of one or combination of specific over_**‐**_, or under_**‐**_expression of cancer-associated molecules such as various proteins, epitopes, growth factors, cytokines/chemokines, receptor/adaptor molecules or enzymes (e.g., Kras, BCR, PI3K, CD11, CD22, Myc, BRCA2, ALK, IL-10, IL-12, p53, p27, p70, MAPKs, TKIs, VEGF, EGF), identified in the molecular tsunami of site-specific cancers [18–22, 27–39, 43–45, 65, 66]. The molecular targets are derived from mutated genetic components (e.g., DNA damage, hypo-, hyper-methylated epigenetic modifications and expression products). While the isolated molecular entities are parts of the highly heterogeneous and chaotic landscape in cancer biology, they should not be considered as ‘target’ for therapy as they have little/no value on their own for translational purposes although they may work in mouse models for the selected conditions and duration of therapy which do not apply to human (see below) [18, 22, 38, 39, 44–46].

Patients with stage III or IV diseases who are treated in clinics, often advance to metastatic stages and develop drug resistance and relapse involving lymph nodes, liver, lungs, bones, and brain resulting in systemic multiple organ failures (MOFs) and damages to vasculature and induction or activation of proteolytic cascade resulting in disseminated intravascular coagulation (DIC) which are most difficult to cure as many physicians experienced [35, 39, 44, 46–49].

In this perspective, attempts were made to briefly review the various therapeutic modalities that have been used for treatment of solid tumors, immunotherapy or safety of pathogen-specific vaccines and the associated cancer financial toxicities for the past several decades [14–18, 22, 29–31, 39–87] (see footnote 3–8).

## Scientific bases for repeatedly failed therapeutic approaches. Molecular false flags and distorted foundations for chemo-immunotherapy

Scientific analyses of data on the repeated failures of the majority of highly publicized and well-funded cancer projects that are claimed as ‘targeted’ therapies, ‘personalized’ or ‘precision’ medicine or the recent trials on ‘immunotherapies’ are rarely reported. The decision makers of such expensive, out-of-focus and fuzzy undertakings seldom consider the life-threatening consequences of wrong and reductionist approaches to drug development for patients and the tremendous economic burden to the society. The irresponsible decision makers of such undertakings, either abandon data on failed outcomes or downplay and ignore the serious consequences of drugs that, at best, postpone patient’s death-sentence for a few months of remission [18–22, 33–39, 44–47]. Once such expensively developed drugs (poisons) failed patients the trials are suspended and soon drug manufactures and decision makers proceed to make minor or major changes to the same protocols (e.g., changes in dosage, route and frequency of drug administration or use of combination drugs). Such strategies are again highly publicized as “new” approaches to cancer drugs through control of media using the same empty promises to justify additional support for recruiting desperate patients in expensive schemes of clinical trials [2–5, 7, 13, 18, 22, 30–38, 41–44].

To better appreciate the issues, according to the NCI updated report (National Cancer Institute Budget Proposal for 2010), the list of major cancer funded studies included the following [reviewed in 44].

i. 12 new drugs or drugs uses (protocol) were approved by FDA;
ii. 348 phase III oncology trials are ongoing;
iii. 861 cancer drugs are in some form of trial process;
iv. 2000-plus clinical trials are accepting children and young adults;
v. 200-plus prevention trials are ongoing and 100-plus screening trials are open.

Since 2010, the above list has grown to include several immunotherapies and numerous pathogen-specific vaccines and cancer trials for recruiting children to clinical trials using the same reductionist and chaotic approaches for young patients (Khatami, manuscript in preparation).

Major scientific reasons for repeated failed therapeutic approaches are outlined below:

- Review of data on molecular targeted therapies shows that the principal scientific reasons for repeated failures are identification of endless genetic mutations in the chaotic molecular environment of cancer [2–4, 17–19, 21, 22, 25–31, 37–40, 44, 45, 65]. Such moving targets on identification of specific and evolving mutations that are bases of the drugs (e.g., potent apoptotic factors or monoclonal antibodies against specific enzymes) that patients are treated with, are highly toxic and cause severe immunobiological and systemic damages to the normal functioning of tissues/organs, rather than being curative for patients. The life-threatening side effects of claimed ‘molecular targeted’ therapies, ‘personalize’ or ‘precision’ medicine include drug-resistance and cancer relapse, anorexia, cachexia, sarcopenia, leukopenia, thromboembolism and metastasis leading to multiple local or distant organ failures (MOFs) and death [19, 22, 28, 37–39, 44, 45]. Therefore, at advanced stages of the disease, the current therapeutic modalities are quite limited in their effectiveness. In addition, the severe and life-threatening side effects of drugs and loss of quality of life (QOL) would cancel out any short-lived benefits from temporary remission of cancer.
- For several decades, numerous circumstantial data, retrospective epidemiological or clinical reports demonstrated that chronic infections, persistent injuries or inflammation induce precancerous state of tissue that increases the risk of many cancers, particularly in aging individuals [37–39, 44, 45]. For example, the pioneering work by Maeda’s group [46–61] demonstrated that infection with influenza virus triggered activation of ROS-generating cascade [e.g., O_2_^·−^ generation via activation of xanthine oxides, in parallel with activation of iNOS (generation of NO), and formation of peroxynitrite (ONOO^−^)] in experimental models of influenza, that causes viral genes mutations and other immune and non-immune modifications. Drug resistance and induction of mutations in chronic infection of hepatitis virus, or *H. pylori*, or *Salmonella typhimurium* infection were also suggestive of the impact of ROS/RNS formation, affecting the genomic structure [46–61]. Numerous other reports also demonstrated a role of immune/inflammatory responses in site-specific tissues leading to initiation and progression of nearly all chronic illnesses including cancer, as well as neurodegenerative and autoimmune diseases [18, 22, 37–39, 44, 45, 51–54, 62–66]. These data support the notion that persistent inflammatory conditions offer powerful chemical, biological and environmental hazards in causing additional genetic alterations at site-specific tissues. Consequently, heterogeneity of such molecular targets and epitopic antigenicity, and distorted molecular components in cancers could render antidote-strategy ineffective and insufficient [18, 22, 36–39, 44, 45, 62–66].
- Recent attempts on extensive trials of cancer vaccines, using viral structures or substructures against several cancers such as cervices, prostate, lung, pancreatic and skin also failed to produce the overall protective clinical outcomes [39–45, 76–78].^7^ While the prophylactic vaccinations could be the most effective and rational medical preventive strategies, their systemic immunity and effectiveness against cancer is debatable. The recent heavily publicized vaccines against human papillomavirus (HPV) such as Gardasil™, or Cervarix™ for prevention of cervical cancers or meningitis vaccines that target young generation, particularly in the United States raise concerns for safety and efficacy of such vaccines [39, 44, 45]. The short or long-term health hazards, efficacy and safety of pathogen-specific vaccines such as virus-contaminated polio vaccines, pneumonia, meningitis, HPV or Swine flu vaccines in the induction of vaccine-(antigen-load) related allergies, autoimmune or neurodegenerative diseases have been raised in a number of reports [39–45, 76–78]. Concerned parents often have to make religion and faith to resist or protest forced vaccination of their school-aged children (Khatami personal communication). The elaborate epitopic targets of cancer seem to have limited prospects and therapeutic cancer vaccination is an area of questionable efficacy for immunotherapy and safety [39–45].
- As recently reported, a closer look at cancer science reveals that highly powered structure (hierarchy) in cancer/medical establishment (system) versus anti-system and chaotic approaches to cancer research and therapy (‘medical/scientific ponzi schemes’) are potent recipes for failed therapeutics that kills patients but generates huge corporate profit [39, 44, 45].
- The recent reports on immunotherapy offer more logical approaches for treating certain tumors (e.g., melanoma, urogenital, breast, non-small cell lung-NSCLC) as they are immunogenic in nature, compared with the identification of endless mutation derived ‘targeted’ therapies that repeatedly failed in patients [22, 36, 46, 62–65, 79–87]. However, carrying out such reductionist studies under the different name of immunotherapy present the same narrow views of cancer biology and are far from being effective for cancer patients. In these studies, little considerations are given to the cellular immune composition of site-specific tissues, the immune-non-immune local or systemic compensatory response mechanisms, the bioenergetics and oxido redox profiles of tissues toward checkpoint inhibition, as well as, the host immune and non-immune interactions with recruited cells and the adverse responses that are observed following therapy [12, 22, 36, 39, 64, 65, 82]. Effective cancer immunotherapy requires systematic understanding of the mechanisms that contribute to the ability of tumor cells to escape and bypass the immune surveillance by induction of decoy receptors, enhanced immune tolerance and loss of mitochondrial function (mitophagy), altered anabolic (growth-promoting) and catabolic (necrosis or growth-arresting) recycling proteins/lipids pathways (autophagy) in tissues. These interdependent complex pathways were defined to be provided through the two biologically opposing arms of Yin (tumoricidal, apoptosis, growth arrest) and Yang (tumorigenic, wound healing or growth promote) pathways of acute inflammation or effective immunity [18, 22, 37–39, 44, 45, 62–65].
- Except for the results of a series of accidental discoveries that were established in 1980 s by Khatami and collaborators [22, 65, 88–92] there is little or no study to identify the early events in the loss of effective immunity that would progressively lead to tumorigenesis and angiogenesis. Analyses of the original data on experimental models of acute and chronic ocular inflammatory diseases are suggestive of the only direct evidence on inflammation-induced time course kinetics of developmental phases of immune dysfunction toward tumorigenesis and angiogenesis. In 2014, Khatami further demonstrated the only evidence on interactions and synergies between host and recruited immune and non-immune cells toward tumorigenesis and angiogenesis [37]. It was suggested that the early events in immune dysfunction could be prevented, reversed or treated [22, 37–39, 45].

In summary, lack of systematic studies on multistep carcinogenesis and the roles that inflammation play in multistep carcinogenesis and concomitant generation of cellular genetic instability and mutations in site-specific tissues are primary scientific factors in failed therapeutics. As recently suggested [22, 37, 38, 46–52, 57–65] accumulation of ROS/RNS could significantly contribute to the impaired mitochondrial function, changes in bioenergetic that are required for maintenance of effective immunity or the balance between two highly regulated and biologically opposing arms termed Yin (tumoricidal) vs Yang (tumorigenic) arms of acute inflammation [37]. It should be noted that the effect of ROS/RNS are additional damages on genetic components at random site. In general, the claimed ‘molecular targeted’ therapies are potent apoptotic factors that would initially inhibit one or a combination of specifically designed growth factors which temporarily cause ‘remission’ or growth-arresting effects on tissues [22, 37–39, 62–66]. However, such drugs would induce an ‘immune tsunami’ or ‘cytokine storm’ throughout the body that destroy the structural integrity and function of vital organs such as the liver, kidneys, bone, muscle and vasculature with life-threatening side effects such as drug-resistant and cancer relapse, cachexia, sarcopenia, thromboembolism, often resulting in MOFs and death [18, 22, 62–65].

## Chemotherapeutic approaches using low molecular weight (LMW) agents: indiscriminate drug-distribution to normal and cancerous tissues

The standard or classic cancer chemotherapy, using low molecular weight (LMW) drugs such as mitomycin C, doxorubicin, methotrexate alone, or even in combination with other drugs for treating solid tumor have not been successful. The toxicities of such drugs often distribute indiscriminately throughout the body with minor tumor-selective accumulation. In addition, except for preferential accumulation of doxorubicin in cardiac tissue, majority of such LMW agents produce systemic toxicity that damages various normal organs/tissues [33, 34, 54, 93–96]. Further increase in the drug dosage is not possible since the dosage level is already at or near their maximum tolerable levels as adverse effects would appear at higher dose. The drug-induced systemic toxicities, in all likelihood, are due to the severe damages to the functional and architectural integrities of tissues such as biophysical, bioenergetics, mechanical organizations and physiology of vital organs leading to significant destruction (suppression) of immune system including damages to bone marrow regenerative processes. The overall toxicities of such drugs on the metabolic and detoxification processes could progressively lead to severe damages to the function of normal organs such as the kidneys, liver, and heart, and it could further involve in coagulopathy and peripheral neuronal toxicity, as well as induction of diarrhea and bleeding.

It should be noted that with the exceptional effect of chemotherapy on seminoma as solid tumors, the classic anticancer drugs such as vinblastine, etoposide, bleomycin, adriamycin, *cis*-platinum, etc., are yielding more than 40–50% responses [84]. While the basis for this remarkable response is not clearly understood, focusing on such approaches may provide better direction for future drug development. Also the effect of BCG with combination of doxorubicin for bladder cancer has been accepted with response rate of more than 50% [79, 97, 98].

The effectiveness of these drugs perhaps is due, in part, to their influence on interdependent growth pathways of phosphatidylinositol 3-kinase (PI3 K)/AKT/mTOR, mammalian target of rapamycin and/or the suppressive effects of interleukin receptor activated kinase-M (IRAK-M) that cause induction of tolerance and growth promotion [22, 37–39, 65]. In addition, drug-induced increased immune suppression in patients facilitates cancer cells to further escape the immune system, resulting in enhanced growth promotion and cancer relapse and metastasis. The adverse effects of erythrocytopenia are often treated with erythropoietin. However, concerns on the induction of thrombosis cannot be ignored. Alternatively, red blood cell transfusion or iron supplement are used to treat erythrocytopenia [2, 3, 70]. Although, leukocytopenia are reasonably treated with granulocyte colony stimulating factor (G-CSF), other drug-induced systemic complications are difficult to control. Quantifying and understanding the molecular/cellular bases of drug toxicity in vivo such as anorexia, cachexia, sarcopenia, bone-marrow suppression, fatigue or weakness, diarrhea, discomfort and pain are yet to be defined as these complications are as important factors in the induction of MOFs and increased morbidity and mortalities in patients, particularly at the progressive stages of the disease [2, 3, 18, 22, 36, 37, 43].

## Targeting genetic mutations in site-specific solid cancers that produced repeatedly failed outcomes while generated huge corporate profits

Molecular target drugs created great business motives for drug industry to focus on them in the last six decades. After revealing extremely high incidence of mutations in solid cancer (Table 1), very little scientific rationale has been presented for developing such costly molecular target drugs that are based on identification of too many evolving genetic mutations in the chaotic cancer environments. Use of fashionable words such as ‘targeted’ therapies, ‘personalized’ or ‘precision’ medicine are attractive for drumming up the support of policy makers and the public while highly lucrative for the decision makers [14–19, 21–24, 26–31, 37, 39, 44, 45, 65, 66] (see footnote 3–7).

**Table 1:** Mutation rate in human cancers Adapted and modified from Refs. [10, 25, 35].

Cancer type	Mutation/tumor
Respiratory/lung cancer	200–300
Skin/melanoma	100–200
Esophageal/colon cancer	50–100
Pancreatic, ovarian	30–60
Breast	20–70
Hematopoietic cancer	1–10
(CML/AML/ALL/CLL) rhabdo/myo/sarcoma	1–3

Mutations of tumor cells were based on means of mutation in single patients

(CML/AML/ALL/CLL) Soft tissue/rhadomyoscarcoma

**Fig. 1:**
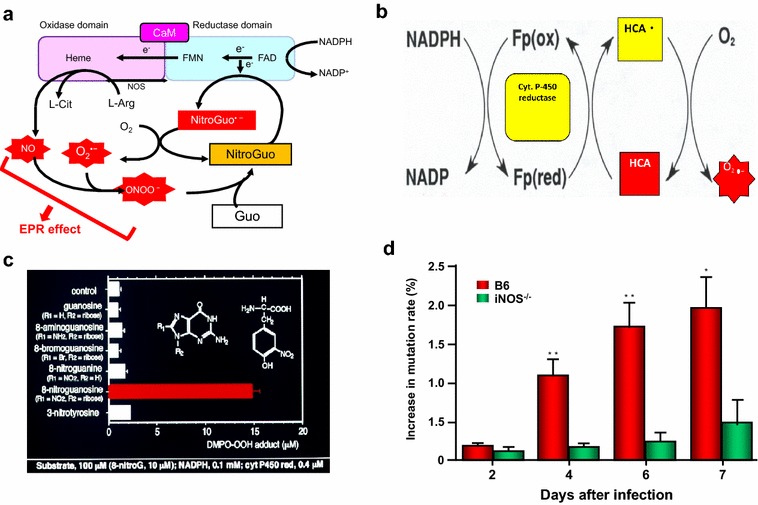
Generation of free radicals by infection and by heterocyclic amine (HCA), and generation of nitrated bases and mutation in Sendai virus via NO. Pathways **a**, **c** and **d** are involved in infection-induced inflamed tissue involving induction of inducible form of nitric oxide synthase (iNOS), and subsequently generation of nitric oxide (NO) and superoxide (O_2_^·−^) and then peroxynitrite (ONOO^−^), which nitrated guanine (→ 8-nitroguanine), and 8-nitroguanosine (NitroGuo), as substrates of NOS or cytochrome c reductase, thereby generation of O_2_^·−^. The total system progressively produces O_2_^·−^, with stoichiometry of greater than 1:1 [51, 100, 108]. **b** Generation of O_2_^·−^ from heterocyclic amine (HCA) in the presence of cytochrome (Cyt) P450 reductase and NADPH, resulting in DNA damage, cleavage and mutation. **c** NADPH cytochrome P450 reductase would generates O_2_^·−^ most effectively from nitroguanosine among other base-modified derivatives [57–61]. **d** Shows the NO dependence of viral mutation. *, **, significant changes in % viral mutations in B6 mice, in comparison with iNOS knockout mice by time. ** statistical significance (< 0.01). See text

One should keep in mind the followings basic biological events that occur in health and disease states of body’s organ systems:

- During normal oxidative metabolism of cell/tissue and function, accidental chemical modifications or genetic errors occur at the rate above 10,000 errors alone in single cell even in the absence of external genotoxic compounds [25–27, 35]. Concerns for cellular mutations that would lead to carcinogenesis often occur when combination of depyrimidination or deamination of cytosine or 5-methylcytosine adenine, guanine and related oxidation damages are at rates that are much higher than 10,000 base per cell per day [27].
- In general, chemical carcinogenesis or mutagenic chemicals interact with DNA or cross-link with segments of DNA, and directly impair DNA replication. Maeda’s group found that chemical carcinogenesis generates ROS or RNS via P-450 related enzymes (e.g., cytochrome P-450 reductase). In this system nitroguanosine acts as substrate to cytochrome b5 reductase or other NADPH reductase-like enzymes (including NO synthase) and generate O_2_^·−^, that further trigger activation of NO synthesis, leading to generation of ONOO^−^ (peroxynitrite) for effective generation of DNA nitrateguanine that would amplify reaction mechanisms (Fig. 1) [49, 56, 57].

In general, chemical carcinogenesis or mutagenic chemicals interact with DNA or cross-link with segments of DNA, and directly impair DNA replication. Maeda’s group found that chemical carcinogenesis generates ROS or RNS via P-450 related exnymes (e.g., cytochrome P-450 reductase b. In this system nitroguanosine acts as substrate to cytochrome b5 reductase or other NADPH reductase-like enzymes (including NO synthase) and generate O_2_^·−^, that further trigger activation of NO synthesis, leading to generation of ONOO^−^ (peroxynitrite) for effective generation of DNA nitrateguanine that would amplify reaction mechanisms (Fig. 1) [49, 56, 57].

Figure 1 represents that NO-dependent viral mutant-formation per 500 plaque in B6 mice, showing green fluorescence protein (GFP)-encoded with Sendai virus infection, resulted in increase of nonfluorescent viral plaque in the lung. This event was compared with iNOS-knockout mice (Fig. 1d) [51, 59]. In addition, Maeda’s group demonstrated similar superoxide generation from highly potent mutagenic heterocyclic amines [99–101]. The observations further support the endogenous generation of ROS and RNS, in addition to direct intercalation with DNA and damages to other metabolic and bioenergetic pathways (see above) [22, 45, 65–69, 71].

The following are highlights of multistep carcinogenesis and current treatment approaches to cancer in experimental and clinical studies:

- The process of carcinogenesis with evolving mutations at multi-stages of cell growth often take anywhere between 10 and 30 years in human before taking over the machinery of dysfunctional immunity. Oxidative stress during aging process that would lead to immune dysfunction could cause generation and accumulation of unrepaired genetic alterations leading to accelerated cell growth. As recently described, cancer cell enhanced growth requirements are satisfied under loss of balance in Yin–Yang of immunity that are associated with differential bioenergetic requirements from mitochondria for oxidative phosphorylation leading to mitophagy and autophagy and hypoxic conditions. The enhanced activities of glycolytic pathways for inefficient energy production (ATP) facilitate growth pathways (tumorigenic or Yang) of immunity [22, 37, 38, 44, 62–65].
- Advances in DNA sequencing technologies indicate that the average patient with site-specific solid tumors such as lung cancer, would have non-synonymous 200–300 mutations per tumor in single patient, while patients with esophageal, breast or colon cancer had somewhere between 50 and 500 mutations per tumor (Table 1) [10, 22, 25–27, 35]. Consequently, making decisions on such evolving high rates of mutations in human solid tumors make these approaches fraudulent (‘molecular false flags’) and irresponsible as evident from the high failure rate outcomes of ‘molecular target’ therapies [18, 22, 36–38, 44, 65]. The claimed molecular target drugs that aim at one or two specific mutations of growth factors, receptors, or enzymes, whether or not the mutations are at “driver seat” at the time they are identified would maximally have 1–3% chances of therapeutic success [29–34, 67, 68]. In addition, such incredibly worthless projects totally dismiss the biological compensatory molecular events of body [18, 22, 33–38]. For example, clinical trials using combination of two inhibitors of EGFR for treating colon cancer did not improve the efficacy compared with single agent, and they are not remarkably different from treating with conventional LMW drugs (shown above). Therefore, it is reasonable to conclude that molecular targeted drugs based on identification of one or few mutated genes or their expression products in the chaotic molecular landscape of cancers (cancer molecular tsunami) would produce very little to benefit the patients. It is not surprising that the outcomes of such expensive undertakings have failure rates ranging between 85 and 95% while causing life-threatening side-effects for patients and draining resources [4–6, 9, 10, 18, 22–29, 33, 34, 37–39, 63–65]. Analyses of similar data on cancer targeted therapies that apply combination drugs such as dasatinib, gemcitabine or debrafenive (debrafenib, or Tafinlar) alone vs. debrafenive + trametinib (Mekinist), for treatment of advanced biliary tract or lung cancers or metastatic melanoma show improved progression-free survival of only few months (8.8 v 9.3 mons or 11.4 v 7.3 mons) while the agents cause serious side effects. These are examples of marginal effects that are economically very costly with tremendous patients suffering [12, 14, 16, 18, 21, 22–25, 70].
- The inherent and diverse compensatory mechanisms of immune and non-immune systems (e.g., vasculature, metabolic and neuronal pathways) in patients treated with specific growth factor inhibition could induce expression of other growth factors locally and/or systemically that would lead to anemia, cancer relapse and metastasis [6, 18, 22, 34–39, 45, 48, 52, 62–65, 70]. Preliminary observations in experimental model of mouse tumors demonstrated that blocking VEGF by antibody caused suppression of tumor growth. However, as treatment with antibody discontinued, tumor growth resumed at similar rate (Maeda et al., unpublished data) suggesting antibody requirements to continue for unlimited period and long-term results may not be beneficiary anyway. Hyper-mutations occur more commonly and frequently in solid tumors, compared with soft tissue cancers and hematopoietic cells (Table 1) [5, 10, 17, 22, 25–27, 35–37, 45]. The latter has a very limited number of mutations and thus respond with higher degrees toward drugs such as Gleevec (imatinib), an inhibitor of protein tyrosine kinase for treating chronic myelogenous leukemia. However, even Gleevec-treated patients suffer from drug-resistant as the consequence of DNA mutations in the treated host at later stages. Recently many drugs developed for Gleevec-resistant patients have considerable success. Although it is an endless game but worth to pursue for better therapeutic for ultimate cure. While these efforts to control the drug-resistance to Gleevec may be encouraging, major motives behind such efforts, seem economical [5]. Furthermore, mogamulizumab, a monoclonal antibody against adult T cell leukemia/lymphoma (ATL) was reported much less effective, compared with imatinib. Currently, the effectiveness of mogamulizumab that is used in combination with conventional anti-leukemic agents makes interpretations of its true efficacy difficult [80]. Similarly, agents such as ipilimumab, that inhibit CTLA-4 for melanoma treatment, and nivolumab that inhibits PD-1 used for treating non-small cell lung cancer, melanoma and renal carcinoma have limited success (20–30% response rate) although drug-induced autoimmune diseases is a major concern [22, 36, 44, 76–79, 81–83].

Therefore, correction of genetic errors and mismatches are normally required for adequate molecular repair function at DNA and/or miRNA levels that also influence post translational modifications throughout life. It is anticipated that if the number of chemical modifications on genome were to be excessive than normal under such conditions as exposures to infective agents, chronic inflammation, environmental, chemical or biological hazards, as well as pathogen-specific vaccines or drug-induced toxicity, particularly during aging, accumulation of defective cells and proteins (e.g., cancerous cells, non-functional proteins, senescent cells) create ‘antigen over load’ that would retard effective immunity, to varying degrees, leading to altered immune response profiles [18, 22, 27, 37–39, 44, 45, 48, 56, 62–66]. As detailed elsewhere, sustained oxidative stress and loss of balance in Yin and Yang of effective immunity could promotes accumulation of molecular errors in tissues and increased damages to genomic stability [22, 44, 45, 52–66]. Oxidative stress-induced accumulation of genetic errors would lead to expression and co-expression of growth and apoptotic factors in susceptible tissues and create an ‘immune tsunami’ that further skew and alter bioenergetics, metabolic, hormonal and neuronal activities in susceptible tissues toward multistep carcinogenesis [18, 22, 62–65].

In summary, the designs of effective cancer clinical immunotherapeutic studies await acceptance of decision makers in cancer community that the inherent immune (cancer) surveillance that was recently defined as the balance between dual properties of Yin (tumoricidal) and Yang (tumorigenic) arms of effective immunity [18, 22, 44, 45, 62–65]. When immune surveillance loses its ability to arrest the growth of oncogenic (defective) cells, cancerous cells progressively and continuously mutate throughout multistep developmental phases of tumorigenesis, carcinogenesis and angiogenesis in susceptible tissues. The results would be progressive expression and co-expression of mismatched and unresolved growth-arresting (Yin, or tumoricidal) and growth-promoting (Yang, or tumorigenic) factors in the immune-responsive tissues (e.g., epithelial-mesenchymal, stroma, vascular endothelial). Unresolved inflammation would facilitate immune evasion and growth promotion of such cells/tissues toward the induction of neoplasia, pre-cancer polyp-formation, cancer, angiogenesis and metastasis [18, 22, 37–39, 44, 45, 62–65].

## Cancer immunotherapy: better logics, same reductionist approaches: controversial understanding of immunity and inflammation

Over the last few decades, cancer immunotherapy, including stem cell transplantation have emerged as the choice for curing cancer on the assumption that cancer cells possessing one or more new antigenic epitopes that could provoke immunological responses, similar to those of immune surveillance in normal host [5, 16, 22, 33, 34, 36, 65, 73, 74]. However, there is no dispute that cancer patients are immune compromised, to varying degrees [5, 16, 22, 33, 34, 52, 62–66, 83, 85–87]. The early approaches on cell-dependent immunotherapies were reported about 40 years ago in mouse tumor models utilizing iv infusion of in vitro activated cultured T cells or LAK cells into the host [85–87]. In these experimental settings, the treatment was successful only when the number of effector cells (E), that is cytotoxic T-cells (CTC) and natural killer cell (NKs) was 30- to 50-fold greater than the number of target tumor cells (T); that is, an E/T ratio of 30 or more was required for tumor regression. However, tumors in human frequently weigh 5–10 g or more. Therefore, it will require 150–300 g of activated T- or NK-cells for infusion. Such approach is therefore unrealistic for treatment of cancer patients. Although this treatment has not been approved by Japanese National Health Insurance, it is still performed in Japan and perhaps other cancer treatment centers around the world.

In cancer immunotherapy, adaptive and innate immune cells such as cytotoxic T cells (CTCs), natural killer cells (NKs) and dendritic cells (DCs) are applied to target T- or B-cell surface receptor molecules with the goal to treat site-specific cancers [22, 64, 66–71]. However, actual success in such approaches requires fundamental understanding of their use and identification and resolution of the current biological gaps that hinder effectiveness of treatment. The important knowledge gaps include identification of composition of host/target immune and non-immune cells, interactions and synergies between host and target tumor cells and understanding of the local and systemic responses that would be involved in specific treatment modalities [18, 22, 39, 44, 45, 65]. One should keep in mind that the outcomes of treatment methodologies using antibody-like molecules that mimic T-cell receptors (TCRs) on host T cell surface proteins that would suppress or arrest the growth; or applying lymphocyte-activated killer cells (LAK) may be different in different site-specific tissues. For example, lung airways, gut-associated lymphoid tissues (GALTs) or conjunctival-associated lymphoid tissues (CALTs) have immunological features that are different from those in liver, stomach, pancreas or non-muscle bladder tissues that potentially contribute to unsuccessful treatment or drug delivery technologies [18, 22, 37–39, 65, 66, 89, 93]. As such, the tremendous knowledge gaps on cellular compositions of target tissues and interactions, or synergies between host tissue and treatment options are likely to limit the effectiveness of such approaches [18, 22, 37–44, 62–65]. Furthermore, jury is still out on the outcomes and effectiveness of stem cell therapy and bone marrow transplant that are used for treating myelocytic leukemia on patients pre-treated with whole body radiation to destroy majority of mutated blood cells. It should be noted that clinical effectiveness of using the overall immune activation by application of bacterial cell components (i.e., BCG) into the urinary bladder tract for bladder cancer seems a more logical approach as a worldwide established method [79, 97, 98]. However, even BCG has its adverse biological effects [22, 28, 39, 42–45, 97, 98].

## Problem of liposomal and micellar drugs. Controversies in stability and drug release from liposomal or micellar complex of antitumor drugs in tumor accumulation

Nanoparticle tumor targeting or delivery is based on the enhanced permeability and retention (EPR) effect [94–115]. EPR effect is a hallmark for targeted drug delivery of biocompatible nanomedicine or macromolecular drugs in tumor tissue [53, 96, 99, 103–109]. The effect can be observed in both primary and metastatic tumors. The EPR effect can be visualized in vivo tumor models or human tumors [99, 108]. The EPR effect reflects pathophysiology of solid tumor including defective vascular architecture, upregulated neoangiogenesis and excessive production of various vascular mediators. It is noteworthy that these factors are common immune disruptors and contribute to the immune dysfunction.

**Fig. 2:**
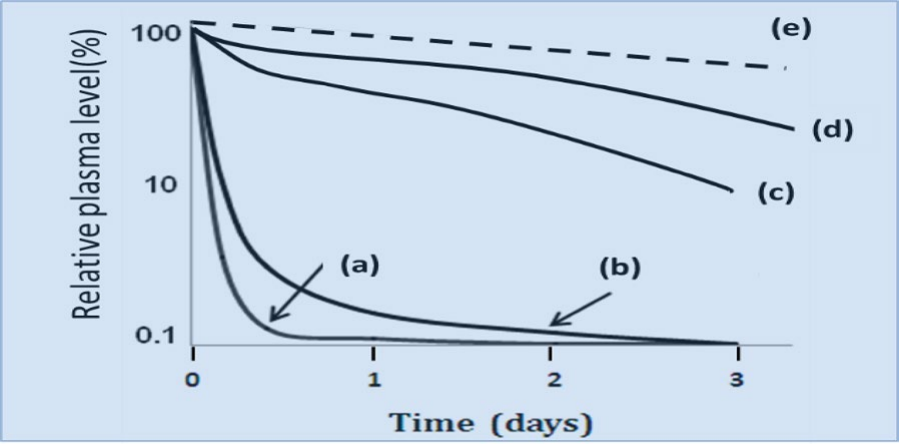
Schematic representation of plasma concentration of different molecular size drugs [33, 34]: a low-MW free drugs (e.g., doxorubicin, DOX) and b–e their polymer complexes. The drug concentration in plasma after i.v. injection of low-MW drugs decreases rapidly (a). Representative polymer conjugates, micelles, and the liposomal drug (DOX) complex remain in the plasma at higher levels (b–e). However (b) shows a micellar drug of non-covalently encapsulated low MW drug which burst rapidly. Thus, no therapeutic benefit due to the EPR effect as its stability is too poor; (c) a styrene-co-maleic acid (SMA)-polymer covalent conjugate having better relative stability [103, 105]; (d) a more biocompatible polymer (HPMA) of pirarubicin conjugate [34]; (e) highly stable and biocompatible liposome complex such as Doxil^®^, showing high concentration in plasma for long period. This stable liposome complex is a pegylated stealth liposome. However, it is too stable and thus little drug release even after reaching to the target tumor, and thus only a limited therapeutic effect. Nano-size drugs (c–e) of high biocompatibility, having long plasma half-lifer, are advantageous for tumor selective targeting because they can utilize the EPR effect [33, 34]

Evaluation of some drug encapsulated liposomes and micellar nanoparticles reveal another example of failed attempts in cancer chemotherapy. Nanotechnology-based nanomedicine has been the focus of great attention in the past couple of decades. Initially, liposome particles presented the poorest outcomes in the pharmacokinetics because of little considerations of the rapid clearance and removal of nanoparticles by phagocytic cells. However, current methods of attaching biocompatible polymers such as polyethylene glycol (PEG) to the surface of particles potentially protect them against this problem. However, in the in vivo setting, it is important that drug-encapsulated liposomes or nanoparticles remain stable and intact enough to reach to the target tissues without disruption of particles or micelles on its way to reach cancer clumps. Otherwise, the active component of LMW drug would often leak out from such particles during circulation and subject to rapid clearance by urinary tract or lymphatic channels, as well as potential decomposition by the liver and the bile. The possibility that particles would burst before reaching the target make such drugs to lose effectiveness while producing adverse effects similar to the parental LMW drug given iv as shown in Fig. 2a, b. In contrast, rigid or sturdy structures of stable particulate drugs such as Doxil^®^, a pegylated liposome-containing doxorubicin (DOX) are too stable and exhibit poor active-drug-release at the tumor tissue or reaching the tumor while resulting poor clinical outcomes [33, 110–112]. As demonstrated in Fig. 1b the iv injection of unstable micellar drug-complexes will be physically disrupted during circulation causing rapid release of the free drug into plasma with no time to achieve EPR effect, which is a time-dependent process. In contrast, covalently linked nanodrugs or micellar drugs have better plasma stabilities in vivo. Figure 1, demonstrates range of plasma concentrations of LMW free drug, such as free DOX or pirarubicin (THP) in respective polymer complexes in vivo.

The use of micellar drug (e.g., NK-911) (Fig. 1b) failed at an early clinical stage due to its insufficient stability, as it bursts too rapidly; losing nearly 50% of its concentration within 1 h after iv injection, and producing no benefit of the required EPR effect [113]. The same logics apply for another biocompatible polymer DOX-conjugates, i.e., HPMA polymer-DOX conjugate (PK1), ~30 kDa molecular size, which failed to produce adequate circulation time required for EPR effect [93–95, 103, 104, 107, 110]. In general, polymers with apparent molecular weight of less than 40 kDa would be too small to produce any effective EPR effect for tumor targeting.

Another example of confusing outcome is a drug designed for macromolecular size, based on the EPR effect for tumor-selective accumulation. In this approach, the miceller agent, fluorescein isothiocyanate (FITC) is covalently linked to a polymer-carrier, while the micelle also contained non-covalently encapsulated candidate-drug (tritiated paclitaxel [PAX]) [99, 104, 107]. The in vivo results showed accumulation of PAX at tumor site was close to null. The non-covalently encapsulated low MW PAX could leak out rapidly from the micelles in the presence of NaCl or blood. However, had paclitaxel covalently linked to the polymer it would have selectively accumulated in the tumor site, as seen in FITC conjugated polymer-chain as the proof of the EPR effect [102, 107].

Therefore, designing effective macromolecular drug complexes requires considerations to include that the selected drugs are stable enough and possesses sufficient biocompatible property, with effective tumor accumulation by EPR effect of the targeted tumors. After delivery in tumor tissue, appropriate drug release need to be incorporated in such drug-complexed nanoparticles [33, 34, 53, 102].

In summary, despite decades of enthusiasm for nanomedicine including liposomal, micellar and polymeric drug complex, there are several problems that need to be addressed. To be effective, such molecular complexes should possess special features, including:

- Retain at high levels in plasma for adequate duration (several hours to a few days) having suitable biocompatibility to be utilized for EPR effect;
- Molecular weights (MW) of macromolecules be above 40 kDa (above renal threshold);
- Complexes would be capable of clearance by lymphatic system in normal tissues, in contrast to cancerous tissue; and
- Complexes capable of extravasations at the tumor’s ‘leaky’ vasculature (angiogenic) sites while allowing adequate liberation of free drug (AP’s) at tumor site, via potential accessible or up-regulated membrane transporter systems (cell-uptake) on tumor cells [33, 34, 102].

Furthermore, there are great differences on cellular uptake rates of different low MW drugs. For example, free pirarubicin (THP) exhibits over 30- to 100-folds higher cellular uptake into tumor cells (pancreatic SUIT-2) compared with free DOX, although both belong to the anthracycline family in which a specific transporter system (e.g., glucose transporter) is highly up-regulated for THP uptake in some tumor cells [95, 102, 114]. Therefore, application of polymer-THP-conjugates seems more advantageous compared with polymer-DOX-conjugates.

## Problems with cancer drug screening and safety in rats and mice: limitations for clinical efficacy in human

Details of the problematic issues in cancer drug discovery and screening methods using experimental mice or rats models of site-specific tumors have been recently reported [22, 44, 115–121]. The major concerns on drug screening are safety and therapeutic efficacies, as well as ethical and financial considerations of decision makers who apply the results that are produced in small animal models in clinical trials to test various anticancer agents in patients which repeatedly failed. The principal concerns with the use of anticancer drugs in clinical trials are briefly discussed below:

- Traditionally, drug development for chronic diseases (e.g., diabetes, hepatitis C, malaria or HIV/AIDS) used chimpanzees as experimental models of human diseases and for drug evaluation purposes. These primates are genetically, behaviorally and biologically the closest animal species to humans. However, in the last few decades, nearly all experimental models of cancer drug screening, safety and efficacy evaluation are performed in lower animals such as rats and mice. The drug screening, efficacy and toxicity of candidate drugs, e.g., monoclonal antibodies against specific growth factors, inhibitors of receptor molecules or kinases, are performed by nu/nu genetic engineered animals, primarily in mice, tissue cultures, or in test tubes, but usually not in chemically-induced autochthonous models. Consequently, as expected the pharmacokinetic parameters or compatibilities of the drugs tested in lower animals are vastly different from those in cancer patients with regard to time scale and immunobiological response profiles and tolerance [22–24, 44, 115–117, 121]. For example, drug screenings are routinely tested in mouse peritoneal leukemia L1210 and P388 models. In such studies, tumors are implanted intraperitoneally (ip), and the drugs also administered via the same route. In such cases, a given drug is likely to be readily accessible to tumor cells in the peritoneal cavity. Under these conditions, pharmacological properties of drugs such as plasma level, tissue distribution, inactivation or clearance from the liver and kidney, and access to vasculature do not pose any serious problem. Consequently, in the ip (tumor)/ip (drug) system, one might demonstrate the desired immediate drug action in tumor cells. These traditional approaches, although better than screening in vitro tumor-cell-panels, totally ignore and downplay the complexity of human solid tumors with complex cellular, stromal or matrix and vascular architectural features of the tumor microenvironment including the neoangiogenesis and vascular permeability, hypoxia, low pH, induction of altered immune and non-immune response dynamics, various proteases that cancer cells excrete, coagulation and cellular clumps of disorganized adhesion properties of cancer cells.
- The anatomical sites used for implanted tumors in mice and the role that vasculature plays will pose differences in efficacies. Implanted tumors are frequently located in the skin or muscle at early stage of development, and not in the orthotopic sites for the primary cancer sites. As a result, one may reasonably question such tests since the drug access to vasculature in experimentally selected sites (e.g., skin) in mice or rats, differ from those cancers developed in the lung, kidney or liver even in mice, let alone in patients which have complex multi-layered architectural organizations and anatomy [115–117]. It is worth emphasizing that even renal cancer cells or hepatoma cells implanted in the muscle tissue of mice do not possess the same features of vascular network, comparable to the kidney or the liver, respectively. In addition, metastatic tumor models are rarely used for drug screening purposes, although it should be the focus of testing if the purpose of screening is the control of advanced stages of cancer [115–117].
- Another problem is the mouse model itself, which is usually a syngeneic system or nude mouse model, when used for studying human xenograft system. Except for identical twins, there are no syngeneic humans. In the syngeneic mouse model, the human tumors (xenobiotic) usually exhibit immunological compatibility with the host mice. Therefore, a host reaction to a xenobiotic tumor is often absent because the tumor would be immunologically inert. Furthermore, the mice implanted with human xenograft tumors do not react immunologically as do the human tumors. In addition, the time scale for tumor development between human and mice are not comparable at all. The so-called window model, using implanted solid tumor in a confined space, i.e., squeezed between two plates of Lucite, is only applicable for very limited cases as tumor is physically so compressed in a confined space and the physical pressure will be built up as artifact. For these reasons, such tumor models represent artifacts, particularly because the doubling time of rodent tumors is so short that it will quickly and physically saturate the space, hence tumor-induced interstitial pressures will be compressed and are not at all comparable to human tumor growth.
- Experimental mouse tumors grow rapidly; that is not usually the case with human tumors. Implants of 5 × 10^6^ tumor cells in a mouse reach a palpable size in about a week or so, whereas human tumors often take months or years to reach a sizeable tumor. There also are 10- to 50-fold differences in doubling time for tumor growth; a few days in mice, and 30–100 days in human. Therefore, relatively fast release of drug from nanoconstructs or liposomes will be found best rate for drugs in mouse system but not suitable for patients.
- The most common endpoint of drug screening system in mice is prolongation of survival rate but not the cure rate, when compared with control group receiving no drugs, in which all mice in test group would eventually die. Complete cure with anticancer agents; claimed ‘targeted’ therapy, ‘precision’ or ‘personalized’ medicine is rarely known, particularly with metastatic solid tumors. The endpoints of cure rate with longer period of more than 100 days in mice, with no recurrence of tumor rarely seen. Investigators should adapt a model that is comparable to the antibiotic-drug-development for infectious diseases decades ago.

In summary, autochthonous or chemically induced models of breast, colon, or liver cancers may offer more realistic tumor models, compared with transplanted syngeneic tumor models. The drug screening designs have little/no considerations for the effect of drug against metastatic tumors, which is by far the most critically important and formidable stage of disease that spreads to distant sites, often beyond surgical removal, while the primary tumor can often be successfully removed by surgery. In general, using mice model may be somewhat more suitable for drug tests for HIV/AIDS patients, having specific immunological response (e.g., T cells) complications to overcome, when compared with cancer patients with multistep immunobiological, metabolic, neuronal and cellular complications. As detailed in recent reports, cancer patients primarily suffer from the severe loss of effective immunity or the balance between Yin (tumoricidal) versus Yang (tumorigenic) properties of immune system that involve loss of oxidative phosphorylation and bioenergetics in mitochondria (mitophagy), enhanced metabolism of glucose (e.g., Warburg glycolysis), loss of cell contact inhibition and altered architectural integrity of site-specific tissues which are advantageous for parasitic survival of cancer cells [22, 44, 45, 62–66]. As proposed below, the above scientific concerns should be taken into consideration for effective systemic chemotherapeutic approaches that could offer serious hope for treating patients.

## Controversies and bias in conducting clinical trials: over-diagnosis, crossovers and randomization of protocols

Patient eligibility to enter the clinical trials most frequently involves stages I and II of the disease. As recently reported [6–9], stage I or even stage II diagnosis for cancer patients are often over-diagnosed. In general, adverse effects of drugs in healthier population are less compared with those observed in patients at advanced stages of the diseases (stages III and IV). Furthermore, ethical concerns and pitfalls regarding the crossover trials that allow patients to switch from control to experimental arms, for receiving investigational drugs remain a serious problem. A reason is that the adverse effects of previously administered drug could not be readily washed out in the body within a month or so, therefore treating patients with a second drug after 1 month may be more hazardous [7–9, 13–17]^8^. The vast differences and bias in the randomized trials using surgical procedures of site-specific cancers, as well as the biological, pharmacological and intrinsic activities of experimental drugs generated in the body would make such crossover trials senseless, if not harmful for the patients [7–9, (Khatami, manuscript in preparation)]. For example, sunitinib is an inhibitor of VEGF for treating renal carcinoma, while iniparib is an inhibitor of DNA polymerization and synthesis [poly(ADP-ribose) polymerase (PARP)] for treating triple-negative breast cancer. These drugs have very different mechanisms of action [3, 4, 6–10] (see footnote 8). Patients suffering from advanced renal cell carcinoma, initially treated with IFN-α might have improved survival outcomes from crossover strategy with sunitinib, as both drugs have potential additive effects in inhibiting VEGF [14–17]. However, using iniparib in crossover trials is not effective for patients with triple-negative breast cancer [3]. One should keep in mind that in general, PARP inhibitors (iniparib) lack intrinsic value for solid tumor. Such drugs are ineffective for BRCA1 and BRCA2 mutations [3, 13–15]. The goal for treatment with iniparib in crossover trials should be potentiating the activities of traditional ‘backbone’ drug in combination and beneficiary to the patients, but they are not. For example, reports for trials that use combination of gemcitabine plus carboplatin, showed outcomes of progression-free survival of only 3.6 months reported for control arm (almost insignificant, and no cure). Furthermore, analyses of data on the outcomes of crossover trials using iniparib, for its effect on sensitization of temozolomide (bevacizumab) in glioblastoma xenograft or clinical targeted therapies of advanced glioma are inconclusive [3, 14–16, 118–120].

## Photodynamic therapy (PDT). A century-old history and little tangible advancement

**Fig. 3:**
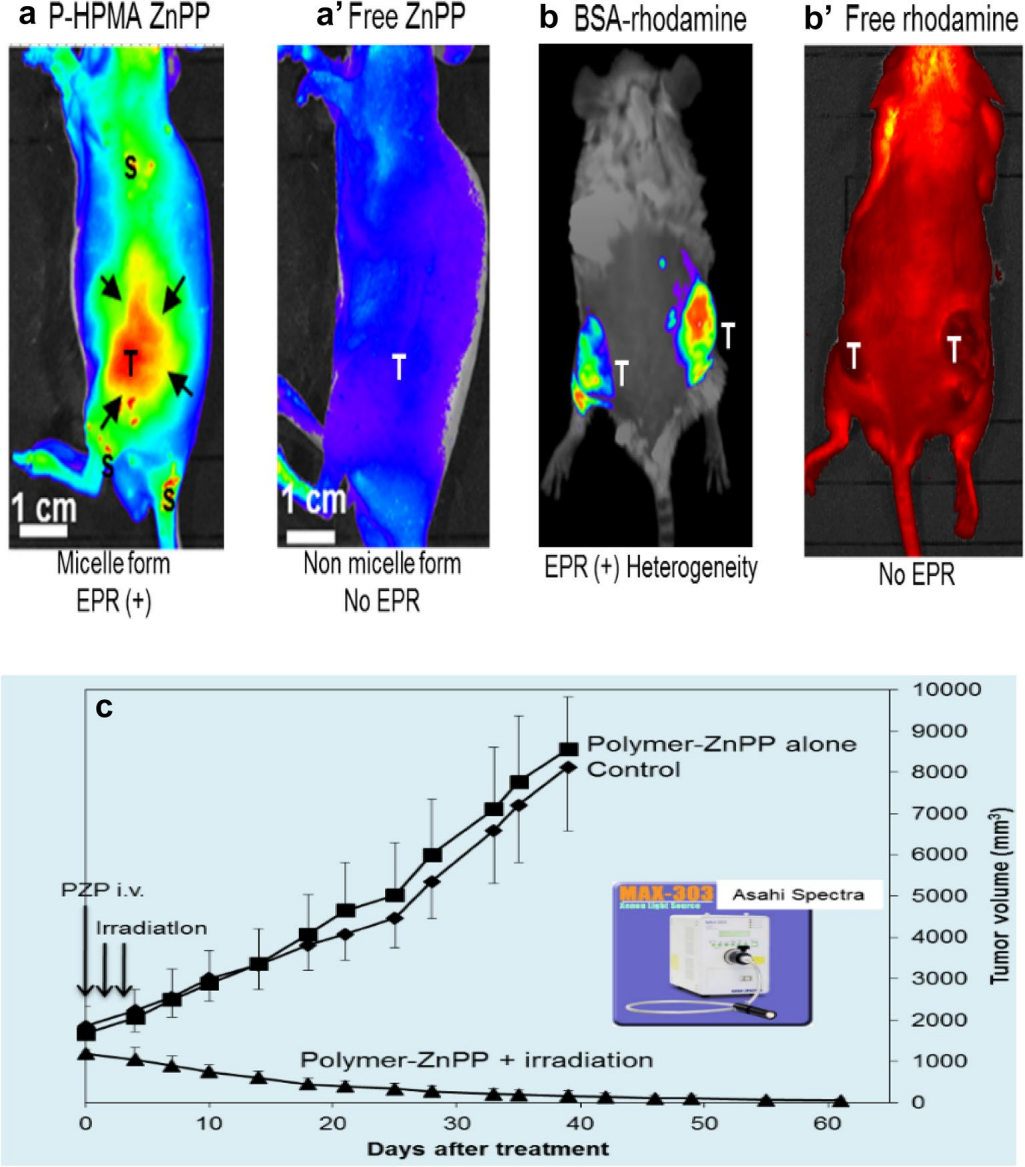
Superiority of macromolecular photosensitizer: **a** polymer (HPMA)-conjugated zinc protoporphyrin (ZnPP) and **b** bovine serum albumin (BSA)-conjugated rhodamine. Fluorescence shows as visible only in tumors (**a**) and (**b**) (T marks). However, when both low MW photosensitizer, free ZnPP (**aʹ**), and free tetramethylrhodamine (**bʹ**) in tumor-bearing mice are injected iv, no tumor selective fluorescence image was visible. Macromolecules, namely polymer-(HPMA) ZnPP and BSA-rhodamine with apparent MWs about 50–70 kDa, respectively, selectively accumulated in tumors, because of the EPR effect, as shown by in vivo fluorescent imaging system; Contrary to above, free ZnPP and free rhodamine, with MWs less than 1000 Da, showed little tumor uptake (**aʹ**,** bʹ**). **c** Demonstrates therapeutic effect of PDT_-_treatment using polymeric ZnPP and endoscopic xenon light irradiation. Tumors used were chemically (diaminobenzene[α]anthracene) induced breast cancer in rats. Polymer-ZnPP alone or light irradiation alone respectively has no therapeutic effect [99, 122] (Figures were adapted from Refs. [99, 122] with permission)

Photodynamic therapy (PDT) for treating diseases is known for more than a century. Indeed, N.R. Finsen received the Nobel Prize in Medicine and Physiology in 1903 for his novel phototherapy of dermal tuberculosis. PDT was expanded to treat cancer about half a century ago, as the use of helium-neon (He–Ne) laser that emits monochromatic light at 633 nm became commercially available. The key component required in PDT is excitation of photosensitizers by appropriate wavelength in the tumor tissue. Most of currently used photosensitizers are derivatives of tetrapyrrolic compounds [121–124]. They require excitation-light around 400–450 nm for optimal effects [121–124]. For cancer treatment, penetration of light (400–500 nm) into cancer tissue is a prerequisite to generate singlet oxygen (ROS). The currently applied He–Ne laser light sources for PDT fail to fulfill the basic principle of spectroscopy for crucial points:Commercial photosensitizers for PDT such as Laserphyrin^®^ and Photofrin^®^ have Soret band of absorption range that produce both intense fluorescence and singlet oxygen (^1^O_2_). However, excitation by He/Ne laser, which emits only at 633 nm, but does not emit at wavelength of 400–450 nm, thus not satisfy the optimal spectroscopic requirements for most efficient generation of singlet oxygen for effective therapy [122]. It should also be mentioned that not all tissues, particularly cancer tissues, are similarly loaded with heme components, like in the normal liver, spleen and blood. When the tissue surface of, for instance, breast cancer is observed visually, or colon cancer by endoscope, the solid tumors exhibit no reddish appearance. As a matter of fact, when we used xenon light of 400–450 nm range directly over the breast cancer it did penetrate sufficient dose of light into the breast cancer in rats, and cancer was completely eradicated (Fig. 3c) [99, 108, 122, 123].As described above for EPR effect, the currently used photosensitizes use molecular weights less than 1000 Da [121–124]. Thus upon iv infusion, they are distributed nearly indiscriminately throughout the body [93, 94, 96, 103] providing no EPR effect and little tumor selectivity. Figure 3a, b represent results of macromolecular-model compound of photosensitizers for tumor selectivity in comparison with low MW counter parts. Using low MW photosensitizer, while producing no remarkable antitumor effect, the patients are advised to avoid exposure to ambient daylight as it is expected to damage the skin with hypersensitivity reactions of the exposed areas. On the contrary, when polymeric photosensitizer and light source (around 430–450 nm) irradiation were used for rat breast cancer in vivo, it produced clear fluorescent tumor image and significant tumor regression [122–124].

## Prohibitive costs of cancer therapy with repeatedly failed outcomes. Economic impact on medical insurance, and unbearable burden to the society

A serious problem in current cancer chemotherapy involves the cost of care for cancer patients, particularly the astronomical costs of recently claimed molecular ‘targeted’ drug, ‘personalized’ or ‘precision’ medicine with outcome failure rates of 85–95% [5, 22, 37, 39, 40, 44, 65, 66] (see footnote 1−8). While majority of such drugs produced no reasonable benefit to meaningfully extend survival of cancer patients, particularly those with solid tumors, they are tremendously costly for the patients, their families and the public [2–8, 13, 18, 20–22, 24, 32, 37, 39, 44, 65–67, 98, 125–127, 131, 132]. Cancer ‘designer’ drugs cost between $100,000–$1000,000 (USD) per course of treatment. For example, nanomedicine type anticancer agents such as Doxil^®^ and Abraxian^®^ cost on average $5000 per injection, that is about 10 times the cost of the parent drugs (doxorubicin and paclitaxel, respectively), without significant survival benefit. The drug makers’ ‘rational’ is that the complex drugs provide more tolerable toxicity for Doxil^®^ compared with free doxorubicin! [125–127].

In the Japanese National Health Insurance System, all patients are eligible to receive government-approved medications and treatments. However, patients must pay out-of-pocket for all medical and hospital costs, if any unapproved medicines were used in conjunction with ongoing/approved treatment. Thus, patients who use any additional unapproved medications lose all privileges of receiving the insurance benefits, even though the particular procedure could potentially provide the needed therapy with proven benefit. For instance, concomitant use of nitroglycerin together with low MW chemotherapeutic agents, significantly benefits the patients with marginal cost [126–130]. It is noteworthy that currently, the total medical expenditure is near 90% of the Japanese National income revenue in 2012 [131]. The government is faced with decision, either to cut this heavy burden for paying the ineffective therapeutic modalities, or alternatively raise the public income-taxes. In the United States, nearly half of the reported personal filings for bankruptcy are due to high cost of medical care resulted from astronomical cost of drugs, hospitalization, medical procedures and patient care [22, 44, 125–127].

Concerned voices of independent and competent professionals, oncologists and scientists that are raised for seeking the truth in cancer science, on behalf of the cancer-stricken public for changing the directions in cancer research or therapy or safety and unethical motives behind development of pathogen-specific vaccines (e.g., HPV, flu, meningitis) that repeatedly failed cannot be ignored or silenced any longer by policy/decision makers [2, 21, 22, 44, 65, 66, 125, 127]. To lessen the heavy burden of costs, for Japanese complex insurance policies, we recommend that the unapproved but potentially effective and safe drugs should become available to cancer patients. The public insurance system should remain continuing coverage of the cost of those drugs that are already approved and marketed for different indications, while those who are willing to undergo treatment with additional experimental drugs, pay out-of-pocket for the cost of drugs that are yet to be approved. It is anticipated that such methods of payment reduce the cost of care for patients who need additional drugs, while the Japan National Health Insurance System can avoid increased debt. We also suggested that the USA policy makers and medical/cancer establishment to return to ‘common sense’ that our forefathers used to serve the public [22, 39, 44, 66].

## Future perspectives: logical, systematic and cost-effective approaches to cancer research and therapy

Lack of systematic approaches to cancer biology is perhaps the principal reason for the extremely slow progress in understanding cancer science, evidenced by high failure rates in cancer therapy and associated loss of millions of lives and tremendous economic burden to the society. The approaches to drug development that are inhibitors against specific growth factors, receptor-molecules or enzymes and are identified in the chaotic and disordered molecular environments of site-specific tumors or current approaches to pathogen-specific vaccines are considered ‘molecular false flags’ based on false foundation. These worthless schemes remind us the USA congressional debates of ‘building bridges to nowhere’ [18, 22, 37, 65, 66]. Decision makers of such thoughtless approaches totally ignore biological consequents of body responses and the extensive harms that are induced to immunity when patients are treated with combination of total (or partial) body radiation and targeted therapy (‘designer drugs’) [18, 22, 39, 65, 66].

Recent paper by Prasad and colleagues [132] supports our scientific concerns that despite reported reduction in disease-specific mortality, the overall mortality was unchanged or increased. Many cancer drugs would initiate or accelerate other causes of death such as disseminated intravascular coagulation (DIC) and multiple organ failures (MOFs) as the consequences of complications such as extreme fatigue or infections, interstitial pneumonia, acute cardiac arrest or cachexia, often resulting in loss of patients lives. Nearly all other claimed molecular targeted therapies that are heavily publicized and funded, focus on identification of infinite genetic mutations in site-specific solid cancers, produced little, if any, success to benefit cancer patients. Majority of such drugs that often accompany total or partial body radiation therapy produce biological poisons to the already immune-compromised patients. The drugs, not only produce life-threatening side effects, but they are extremely costly for patients and insurance companies.

Below we outline that future systematic approaches to study the amazing complex role of immune disruptors-induced initial immune dysfunction toward multistep carcinogenesis that are intimately associated with angiogenesis (hallmark of tissue growth, hypoxia and altered bioenergetics) offer tremendous opportunities for research and therapeutic considerations [22, 36–39, 56, 65–67, 108, 126–154].

- Modalities that utilize nanotechnologies for tumor-selective drug delivery, based on the EPR effect with full consideration of tumor environments. Utilization of tumor environments for tumor-selective drug accumulation include the lower pH of tumor tissue (1–1.5 units) compared with normal tissue (pH 7.4). In addition, the unique features of upregulated glucose transporter in tumor provide good targets using glycosyl-containing moiety for drug development. Furthermore, hypoxia that is the result of embolized blood flow in solid tumor vessels may be restored by nitro agents or alike, to improve the blood flow and drug delivery. Acidic environment of tumor are suggested suitable site for EPR and cleavage by hydrolytic enzymes (e.g., cathepsin, MMPs, etc.), or spontaneous cleavage of acid labile bonding (e.g., hydrazone, ester bonds) between linker polymers and desired drugs.
- Systematic studies to understand immune disruptors-(oxidative stress) induced initial pathways in developmental phases of immune dysfunction in the direction of multistep tumorigenesis and angiogenesis. Effective immunity was defined as the balance between two highly regulated and biologically opposing arms, Yin (tumoricidal) and Yang (tumorigenic) properties of acute inflammation, an amazingly precise signal communications between immune and non-immune systems. The Yin and Yang events were hypothesized requiring differential bioenergetics at different stages of life, from fetus growth, after birth toward adulthood and aging process or chronic diseases. Unresolved inflammation was described as a common denominator mapping aging process and the induction of ‘mild’, ‘moderate’ or ‘severe’ immune disorders including cancers. Detailed understanding of the loss of balance in tumoricidal (Yin) and tumorigenic (Yang) properties of effective immunity that guards health should be the focus of future studies.
- Details of pathogen-host interactions and immune response profiles in susceptible tissues. We recently proposed that chronic inflammation causes release of histamine at local and distant tissues altering numerous other immune responses and the acid-base behaviors in tissues including vasculature. Histamine was proposed as blue print in the genesis of ‘mild’, ‘moderate’ or ‘severe’ immune disorders including site-specific cancers.

The above logical approaches to therapy and basic research on complex biology of effective immunity are expected to result in the design of cost-effective projects for understanding not only the cancer biology or how to prevent or control (treat) it, but also effective approaches for development of universal vaccines and overall promotion of health. Furthermore, systematic approaches in understanding effective immunity are expected to lay a foundation for minimizing or delaying the onset of nearly all other chronic and preventable diseases for the aging populations around the world [22, 36–39, 43–45, 65, 66].

## Concluding remarks

The focus of this perspective was to assess the limitations of current therapeutic approaches to cancer. We presented scientific analyses of the disturbing data on the outcome failure rates of 90% (±5) on current therapeutic approaches for solid tumors. In the last six decades, only limited success was achieved with drugs such as Gleevec or few other modalities that used for treating patients with hematopoietic cancers and soft tissue or seminoma.

The future logical directions for cancer science and therapy to be beneficiary to the public should focus on restoration of immune surveillance, the body’s protective mechanism for killing cancerous cells. The claimed ‘targeted’ therapies that may or may not extend remission of cancer for a few months should not be accepted any longer as ‘cure’ by oncologists, scientist or patients. These tremendously costly projects totally disregard the suffering and life-altering experiences of patients and their families or caregivers [18, 22, 28, 32, 37, 39, 44, 65, 66, 125–127, 154]. Torturous period of survival overwhelms the benefit of postponing ‘death-sentence’ of patients for few months. It is important to seriously consider that the cost for conducting too many out-of-focus projects including usage of specific detection technologies for ‘targeted’ or ‘designer’ drugs that repeatedly failed patients has increased 340 time in the last 10 years, while accomplished very little. This horrendous view for making profit out of misery of patients can no longer be sustained or tolerated.

Another serious concern in process of drug development, however wrong, is the long processes and delays in obtaining patents, proprietary and approval for new drugs. In most countries, exclusivity of proprietary for marketing a specific drug often guaranteed for up to a decades. We propose abolishing the currently imposed regulations of drug patent system with the goal to accelerate generic drug development and improved access of drugs to patients. Often the industry manages to extend blocking the patented drugs for unlimited time, for maintaining the marketed drug prices at sky high and for high profits.

## Abbreviations

ALL: acute lymphocytic leukemia
AML: acute myelogenous leukemia
BCR: breakpoint cluster region
BRCA: hereditary breast cancer gene
CLL: chronic lymphocytic leukemia
CML: chronic myelogenous leukemia
DIC: disseminated intravascular coagulation
DOX: doxorubicin
dTNFR: decoy receptor of tumor necrosis factor
EGF: epidermal growth factor
EPR: enhanced permeability and retention
HPV: human papilloma virus
HPMA: *N*-(2-hydroxypropyl)methacrylamide
iv: intravenous
LAK: lymphocyte-activated killer cells
LMW: low molecular weight
MCs: mast cells
MMPs: matrix metalloproteinases
MOF: multiple organ failure
NK: natural killer
NO: nitric oxide
NO_2_^−^: nitrite
O_2_^·−^: super oxide anion radical
PAX: paclitaxel
PDT: photodynamic therapy
PEG: polyethylene glycol
PG: prostaglandin
PI3 K: phosphoinositol-3-kinase
PS: photosensitizer
QOL: quality of life
RO: alkoxyl radical
ROO: alkylperoxyl radical
ROS: reactive oxygen species
SMA: styrene-co-maleic acid polymer
mTOR: mammalian target of rapamycin
TCR: T cell receptor
THP: tetrahydropyranyl doxorubicin (pirarubicin)
TLRs: Toll like receptors
VEGF: vascular endothelial growth factor
ZnPP: zinc protoporphyrin

## Footnotes

1 In February of 2012, in a National Cancer Institute (NCI) Board meeting report on cancer therapy admitted that success rate being 15% also, in March of 2012, in a Metabolon conference in Bethesda, Maryland, company professionals reported that 95% of cancer drug developed failed.

2 Medscape December 5, 2011 (Washington DC) reported by Dr. Foji (NCI) 'zero (is) the number of targeted therapies that prolonged survival by 1 year' when compared with conventional treatment.

3 June 1, 2012, E Berger (CNBC program) in an interview with then president of MD Anderson, DePinho confirmed that 95% of cancer drugs for solid tumors fail –http://blog.chron.com/sciguy/2012/06/m-d-anderson-president-goes-on-cnbc-extols-his-own-company.

4 “Is The U.S. Becoming a Police State to Force Mandatory Vaccination?”; also “American Academy of Pediatrics wants a Police-State approach to vaccination”, Health Impact News, September 14, 2016.

5 “Complaint to the European ombudsman over maladministration at the European Medicines Agency (EMA) in relation to the safety of the HPV vaccines”. Letter signed by professionals regarding HPV safety concerns; Nordic Cochrane Centre, Copenhagen, Denmark. October 10, 2016.

6 Robert F. Kennedy, Jr. Exposes New Evidence of CDC Corruption Regarding Vaccines and Autism and related reports on vaccines safety concerns-World of Mercury-accessed from Health Impact News, September 18, 2017.

7 Lisa Stark, Legal Correspondence, on Vaccines—PBS News Hour September 26, 2017.

8 Summary Health Statistics for U.S. Adults: CDC, National Health Interview Survey (2011).
